# Correction: Dense Genotyping of Immune-Related Loci Identifies Variants Associated with Clearance of HPV among HIV-Positive Women in the HIV Epidemiology Research Study (HERS)

**DOI:** 10.1371/journal.pone.0102728

**Published:** 2014-07-08

**Authors:** The PLOS ONE Staff

## Notice of Republication

This article was republished on June 18, 2014, to correct Figure 1, which was missing panels B-D. Please download this article again to view the correct version. The originally published, uncorrected article and the republished, corrected article are provided here for reference.

## Supporting Information

File S1 Originally published, uncorrected article.
(PDF)

File S2 Republished, corrected article.
(PDF)
