# Supplementary material for: Correction: Dense Genotyping of Immune-Related Loci Identifies Variants Associated with Clearance of HPV among HIV-Positive Women in the HIV Epidemiology Research Study (HERS)
Source: PLoS One. 2014 Jul 8;9(7):e102728. doi: 10.1371/journal.pone.0102728 (PMC4087035; doi:10.1371/journal.pone.0102728)
Supplement: File S2 — Republished, corrected article. (PDF) [file pone.0102728.s002.pdf]

PLOS ONE

Dense Genotyping of Immune-Related Loci Identifies Variants Associated with Clearance of HPV among HIV-Positive Women in the HIV Epidemiology Research Study (HERS)

Staci L. Sudenga[1], Howard W. Wiener[1], Caroline C. King[2], Anne M. Rompalo[3], Susan Cu-Uvin[4], Robert S. Klein[5], Keerti V. Shah[6], Jack D. Sobel[7], Denise J. Jamieson[2], Sadeep Shrestha[1]*

1 Department of Epidemiology, University of Alabama at Birmingham, Birmingham, Alabama, United States of America, 2 Division of Reproductive Health, Centers for Disease Control and Prevention, Atlanta, Georgia, United States of America, 3 Division of Infectious Diseases, Johns Hopkins University School of Medicine, Baltimore, Maryland, United States of America, 4 Department of Obstetrics and Gynecology and Medicine, Brown University, Providence, Rhode Island, United States of America, 5 Mount Sinai School of Medicine, New York, New York, United States of America, 6 Department of Molecular Microbiology and Immunology, Johns Hopkins University Bloomberg School of Public Health, Baltimore, Maryland, United States of America, 7 School of Medicine, Wayne State University, Detroit, Michigan, United States of America

Abstract

Persistent high-risk human papillomavirus (HR-HPV) is a necessary and causal factor of cervical cancer. Most women naturally clear HPV infections; however, the biological mechanisms related to HPV pathogenesis have not been clearly elucidated. Host genetic factors that specifically regulate immune response could play an important role. All HIV-positive women in the HIV Epidemiology Research Study (HERS) with a HR-HPV infection and at least one follow-up biannual visit were included in the study. Cervicovaginal lavage samples were tested for HPV using type-specific HPV hybridization assays. Type-specific HPV clearance was defined as two consecutive HPV-negative tests after a positive test. DNA from participants was genotyped for 196,524 variants within 186 known immune related loci using the custom ImmunoChip microarray. To assess the influence of each single-nucleotide polymorphism (SNP) with HR-HPV clearance, the Cox proportional hazards model with the Wei-Lin-Weissfeld approach was used, adjusting for CD4+ count, low risk HPV (LR-HPV) co-infection, and relevant confounders. Three analytical models were performed: race-specific (African Americans (n = 258), European Americans (n = 87), Hispanics (n = 55), race-adjusted combined analysis, and meta-analysis of pooled independent race-specific analyses. Women were followed for a median time of 1,617 days. Overall, three SNPs (rs1112085, rs11102637, and rs12030900) in the *MAGI-3* gene and one SNP (rs8031627) in the *SMAD3* gene were associated with HR-HPV clearance ($p < 10^{-6}$). A variant (rs1633038) in *HLA-G* were also significantly associated in African American. Results from this study support associations of immune-related genes, having potential biological mechanism, with differential cervical HR-HPV infection outcomes.

Citation: Sudenga SL, Wiener HW, King CC, Rompalo AM, Cu-Uvin S, et al. (2014) Dense Genotyping of Immune-Related Loci Identifies Variants Associated with Clearance of HPV among HIV-Positive Women in the HIV Epidemiology Research Study (HERS). PLoS ONE 9(6): e99109. doi:10.1371/journal.pone.0099109

Editor: Carlo Federico Perno, University of Rome Tor Vergata, Italy

Received February 4, 2014; **Accepted** May 10, 2014; **Published** June 11, 2014

This is an open-access article, free of all copyright, and may be freely reproduced, distributed, transmitted, modified, built upon, or otherwise used by anyone for any lawful purpose. The work is made available under the Creative Commons CC0 public domain dedication.

Funding: Genotyping efforts were supported by the Pilot Project Award (PI: Sadeep Shrestha) from the Johns Hopkins Cervical Cancer SPORE (2P50CA098252 PI: TC Wu). The work was supported in part by the NIH Cancer Prevention and Control Training Program (R25CA47888 – fellowship of Staci Sudenga). The HIV Epidemiology Research Study (HERS) was funded through Centers for Disease Control and Prevention cooperative agreements U64/CCU106795, U64/CCU206798, U64/CCU306802, and U64/CCU506831. The funders had no role in study design, data collection and analysis, decision to publish, or preparation of the manuscript.

Competing Interests: The authors have declared that no competing interests exist.

* E-mail: sshrestha@uab.edu

Introduction

HPV DNA is present in 99.7% of all cervical cancer, the third most common cancer in women worldwide, with HPV types 16, 18, 31 and 45 being the most predominant HR-HPV types [1–5]. HPV is a common sexually transmitted infection and the majority of those infected are able to clear the infection naturally and only a small proportion will progress to cervical cancer [6,7]. A persistent infection of a HR-HPV type is considered the most important factor for development of pre-cancer high-grade lesions and progression to cervical cancer [8]; although, it is not a sufficient cause. Further, while cervical cancer is a definitive end to the stages of progression associated with HPV infection, it is important to understand the earlier biological processes of HPV persistence in the host.

The factors that lead to the development of a persistent HPV infection in some women, but not others, remain unclear. The role of host genetics that regulate biological mechanisms of immune response may contribute to the differential responses to infection and HPV clearance among women. To our knowledge, most research on host genetics including genome-wide association studies (GWAS) have focused on cervical cancer as the outcome [9,10], and few have investigated the influence on HPV persistence, the intermediate phenotype to cervical cancer. Even

with the limited genetic epidemiology studies of HPV infection as an outcome, the study designs have been predominantly cross-sectional, comparing genetic variants between women with HPV-positive cervical cancer and randomly selected controls [11,12]. Since persistence of HPV is time-dependent, longitudinal studies assessing HPV over time would be more appropriate to examine the true association. Several studies implicate immune evasion [13–15] involving genetically mediated determinants of the localized cell-mediated immune response in the host to be an important factor in the pathogenesis of HPV infection [16,17]. Specifically, human leukocyte antigens (HLA) variants (*DRB1*13* and *DRB1*1501-DQB1*06*) have been shown to be associated with cervical cancer [10,18] and possibly HPV infection [19]. Further, even among women who naturally clear HPV infection, there are differences in time to clearance suggesting the role of the host immune system. While HPV persistence is necessary for development of most cervical premalignant and cancers, the role of immune-related genes (such as those in the ImmunoChip) are biologically meaningful in relation to viral pathogenesis and may or may not be involved in the development or progression of pre-cancer lesions. The knowledge of immune response will complement our understanding of the pathogenesis of HPV. Our objective was to assess the association of host genetic variants in immune-related genes with clearance of HR-HPV using the Human ImmunoChip in HIV-positive African Americans, Hispanics, and European women.

Materials and Methods

Ethics Statement

Written consent was obtained from all participants (parental written permission was obtained for minorities wherever required) and the study protocols for the parent study and this sub-study was approved by institutional review boards (IRB) at all sponsoring organizations and conformed to human-experimentation guidelines set forth by the United States Department of Health and Human Services. All protocols including the genetic work was finally reviewed and approved by the University of Alabama at Birmingham IRB.

Study Population

Participants from the HIV Epidemiology Research Study (HERS) cohort were included in this study [20,21]. HERS is a multicenter, prospective study established by the Centers for Disease Control and Prevention (CDC) to examine the natural history of HIV in women. Women aged 16–55 with documented HIV status and high-risk behaviors were recruited between April 1993 and January 1995. The exclusion criteria in the parent study were as follows: i) had no identified HIV risk behavior; ii) had risk only by transfusion history or vertically from HIV-positive mother; iii) were not born female (i.e., transsexual); iv) did not consent to the full protocol, including pelvic exam, phlebotomy, and repeated HIV testing and counseling; and v) reported previously having AIDS-defining illnesses. Of the 1,994 women screened, 1,310 (66%) were enrolled in the study (871 HIV-positive and 439 HIV-negative). After enrollment, the core visit included a physical examination with complete gynecologic exam and specimen collection. Blood was tested for CD4+ T lymphocyte cells (CD4+) count and HIV viral load at 6-month intervals.

HPV DNA Detection and Classification

HPV testing was performed on all HERS women at enrollment and every 6 months thereafter using cervicovaginal lavage samples. Viral DNA fragments from cervicovaginal lavage were amplified by using the consensus primers MY09/11 and HMB01 and were hybridized for a consensus probe and for 26 HPV types HR-HPV: 16, 18, 26, 31, 33, 35, 39, 45, 51, 52, 56, 58, 59, 66, 68, 73, and 82 and LR-HPV: 6, 11, 40, 42, 53, 54, 55, 83, and 84 using a chemiluminescent dot-blot format [20]. PCR-based HPV data were classified as a) negative, b) positive for the specific types, or c) positive, type unknown if the sample was positive for the generic probe but not for a specific HPV type. PCR amplification of a human β-globin gene segment was used as an internal control for DNA quality; samples negative for this assay were excluded from analyses.

The analysis was performed in HIV-positive women in HERS who had no cervical treatment and were not pregnant during the follow-up. HIV-positive women with prevalent or incident HR-HPV infection who had peripheral blood mononuclear cells (PBMC) available at the baseline visit and at least one follow-up visit were included in the genetic analysis. In this study, the analyses focused on clearance of HR-HPV infections since these types are most likely to be associated with risk of pre-cancer and cancer of cervix and the models were adjusted for LR-HPV infections in order to account for the correlation between HPV subtypes. For the analysis, type-specific HPV status was assumed to remain unchanged across single missing visits. For women who tested negative for HPV at their last study visit, results were censored at that visit. Type-specific HPV clearance was defined as two consecutive HPV-negative tests to avoid the possibility of false-negative test results.

Genotyping

Genomic DNA was extracted from stored PBMC and genotyped using the Human ImmunoChip, an iSelect HD custom genotyping array (Illumina, Inc.). The ImmunoChip is a custom SNP microarray developed by a consortium of specialists in the fields of immunology and inflammation. The microarray chip contains densely spaced 196,524 SNP variants (5,001 non-synonymous coding, 1,926 synonymous coding, and 4,065 in the untranslated region (UTR)), previously reported from GWAS and candidate gene studies of major autoimmune and inflammatory diseases [22,23]. The genotyping of the samples was processed at two different laboratory facilities, and this was adjusted for in the statistical models.

Immunochip Genotype Calling and Quality Control

Data were analyzed using the Genome Studio Genotyping Module (Illumina, Inc.). The National Center for Biotechnology Information (NCBI) build 36 (hg18) map was used and the normalized probe intensities were extracted for all samples that passed standard laboratory quality-control (QC) thresholds. The Immunochip contains 763 duplicate SNPs and these were checked for concordance. Additionally, 174 genotyping assays failed which left 195,587 SNPs. All SNPs were checked for completeness (by SNP and by subject), rare variants, and deviation from Hardy-Weinberg Equilibrium (HWE) within each ethnic group separately. First, a large proportion of SNP assays failing on an individual may indicate poor quality of DNA sample and thus to avoid aberrant genotype calling, the threshold was set to 90% coverage. All African Americans and European Americans samples met this criterion, but 2 Hispanic samples did not and thus were removed from the analyses. Second, missing genotype data from a large number of individuals indicate poor assay quality for that SNP, and there were 5171 SNPs in African Americans, 5889 SNPS in European Americans and 6131 SNPs in Hispanics that were missing in more than 10% of individuals and were removed from the analysis. Third, there were 71,488 SNPs in African Americans,

73,017 in European Americans, and 68,264 in Hispanics that had a minor allele frequency less than 5% (rare variants) that were also removed in the analysis of each ethnic group, respectively. Fourth, there were 1234 SNPs in African Americans, 442 SNPs in European Americans, and 347 SNPs in Hispanics that deviated from HWE (p-value$<$0.001), and thus were also removed from the analyses. Further, kinship between the individuals was also assessed in KING software [24], and two of the African American individuals were determined to be genetically related. However, one of the individuals had a lot of missing data and the relatedness seems likely due to specimen contamination; this individual was removed from the analyses (removal of both individuals did not change the results of the analysis). There were no major differences with the genotype call rates, minor allele frequency between the two genotyping facilities; however, they were adjusted for in all our analyses. After applying the QC methods above, there were 258 African Americans with 117,694 SNPs, 87 European Americans with 116,239 SNPs, and 55 Hispanics with 120,845 SNPs included in the analysis.

The same QC methods mentioned above for each ethnic group separately were then applied to the combined analysis which contained all three ethnic groups. For the combined analysis of all three distinct race (described below), there were 5190 SNPs removed because of low genotyping calls and an additional 66,536 rare variants ($<$0.05%) across any one ethnic group and were removed; thus, 123,861 SNPs were available for analysis. Since SNPs in the ImmunoChip are mostly in the fine-mapped regions with high linkage disequilibrium (LD), correction for multiple testing was performed based on the effective number of tests which resulted after pairwise correlations between markers, as previously described [25]. Based on this matrix, there were 94,307 effective tests among African Americans, 93,164 among European Americans, and 96,967 among Hispanics that resulted in adjusted p-value corrections of 5.30×10^{-7}, 5.37×10^{-7}, and 5.16×10^{-7}, respectively. Additionally, among the SNPs common in all three ethnic groups, there were 80,510 effective numbers of tests (adjusted p value = and 6.21×10^{-7}).

Statistical Analysis

Cox proportional hazard models with the Wei-Lin-Weisfeld (WLW) extension [26] were used to assess the influence of all SNPs (individually) with clearance of HR-HPV [27]. This approach can simultaneously analyze time to HPV clearance of several types of HPV either at the same or different visits, taking into account possible correlation between the types and also has population-level interpretation [8,26], making it appropriate for epidemiological studies. The WLW model was implemented in SAS using PHREG procedure, selecting the STRATA option to allow different baseline hazards function for each HPV type and robust variance.

To test for potential confounding effects of population stratification in our study cohort, principal component (PC) analysis was performed. Self-reported race (African Americans, European Americans, and Hispanics) was confirmed using clustering as implemented in EIGENSTRAT, and all individuals were confirmed with no apparent population outliers [28]. Hazard ratios (HR) and 95% confidence intervals were calculated for all races (n = 400) combined into one model (referred to as "race-adjusted analysis") adjusting for the first 10 PC values. Hazard ratios (HR) and 95% confidence intervals were also calculated for each race separately (African Americans (n = 258), European Americans (n = 87), and Hispanics (n = 55)) adjusting for the first three PC values. Further, a meta-analysis was using PLINK [29] pooling the results from individual race-specific analyses (referred to as "pooled analysis").

All SNP analyses were conducted using an additive genetic model, adjusting for CD4+ count, which served as a marker for immune status as well as a surrogate for HIV treatment. CD4+ count was adjusted for in the model at all visits when an individual was infected with HPV, which allowed CD4+ count to serve as a time-varying covariate. LR-HPV infections were adjusted for in the model when an individual was co-infected with any HR-HPV type, which also allowed LR-HPV to serve as a time-varying covariate when infection time overlapped. Quantile-quantile (Q-Q) plots of p-values were constructed to evaluate deviations from the expected test statistic distribution. ImmunoChip based Manhattan plots were generated to visualize the results.

Results

The average age at baseline of the 400 HIV-positive women included in the study was 34 years and their median baseline CD4+ count was 426.3 cells/mm^3 [interquartile range (IQR): 262.3–546.1 cells/mm^3]. Women were followed for a median time of 1,617 days (range 324–1729 days). Among the 400 women, there were 1052 HR-HPV infections, and 668 (64%) cleared during follow up. The most common HR-HPV types were HPV18 (n = 99), HPV16 (n = 88), HPV51 (n = 88), HPV58 (n = 83), and HPV52 (n = 79). Of the 668 infections that cleared during follow up, the average time to clearance was 457.9 days (median 322 days). Among the HR-HPV infections that cleared during follow-up, the average CD4+ count was 385.1 cells/mm^3 (median 336.7 cells/mm^3). Among the HR-HPV infections that persisted during follow-up, the average CD4+ count was 251.1 cells/mm^3 (median 212.9 cells/mm^3).

The Manhattan plots (Figures 1A–D) summarize the results from the association between HR-HPV clearance and the SNPs in the ImmunoChip (Q-Q plots - Figures S1A–D). All significant hits based on the K-effective method described above are presented for the three analytical models in Table 1 (race-adjusted analysis, race-specific analysis, and the pooled analysis). Results for all of the SNPs assessed can be found in Table S1. In the race-adjusted analysis, three SNPs that are in LD (rs1112085, rs11102637, and rs12030900) in the *MAGI3* gene were associated with HPV clearance. HIV-positive women infected with HR-HPV that have minor allele A for SNP rs1112085, located on chromosome 1, had a HPV clearance rate 1.51 times ($p = 1.14\times10^{-07}$) higher than those with the G wild type allele, controlling for CD4+ count, LR-HPV infection(s), 10 PCs, and genotyping facility (Table 1). When assessing this SNP (rs1112085) in the three races separately (Table 1), in African Americans and European Americans the minor allele was associated with higher clearance rates (HR = 1.49, $p = 4.07\times10^{-05}$, HR = 1.53, p = 0.01, respectively), while no association was detected between this SNP and HR-HPV clearance among Hispanics (HR = 0.98, p = 0.91). In the pooled analysis using the meta-analysis approach, the same minor allele was associated with higher clearance rates (HR = 1.33, p = 0.03). Likewise, a SNP (rs8031627), located on chromosome 15 in the *SMAD3* gene, was also significantly associated with higher clearance rates in the race-adjusted analysis (HR = 1.53, $p = 8.04\times10^{-07}$) and the pooled analysis (HR = 1.49, $p = 1.78\times10^{-06}$). When analyzed among the separate races, the SNP (rs8031627) was significantly associated with clearance in Hispanics (HR = 1.76, p = 0.0005) and African Americans (HR = 1.44, p = 0.003); a similar hazard ratio was seen in European Americans, but it was not statistically significant (HR = 1.34, p = 0.08).

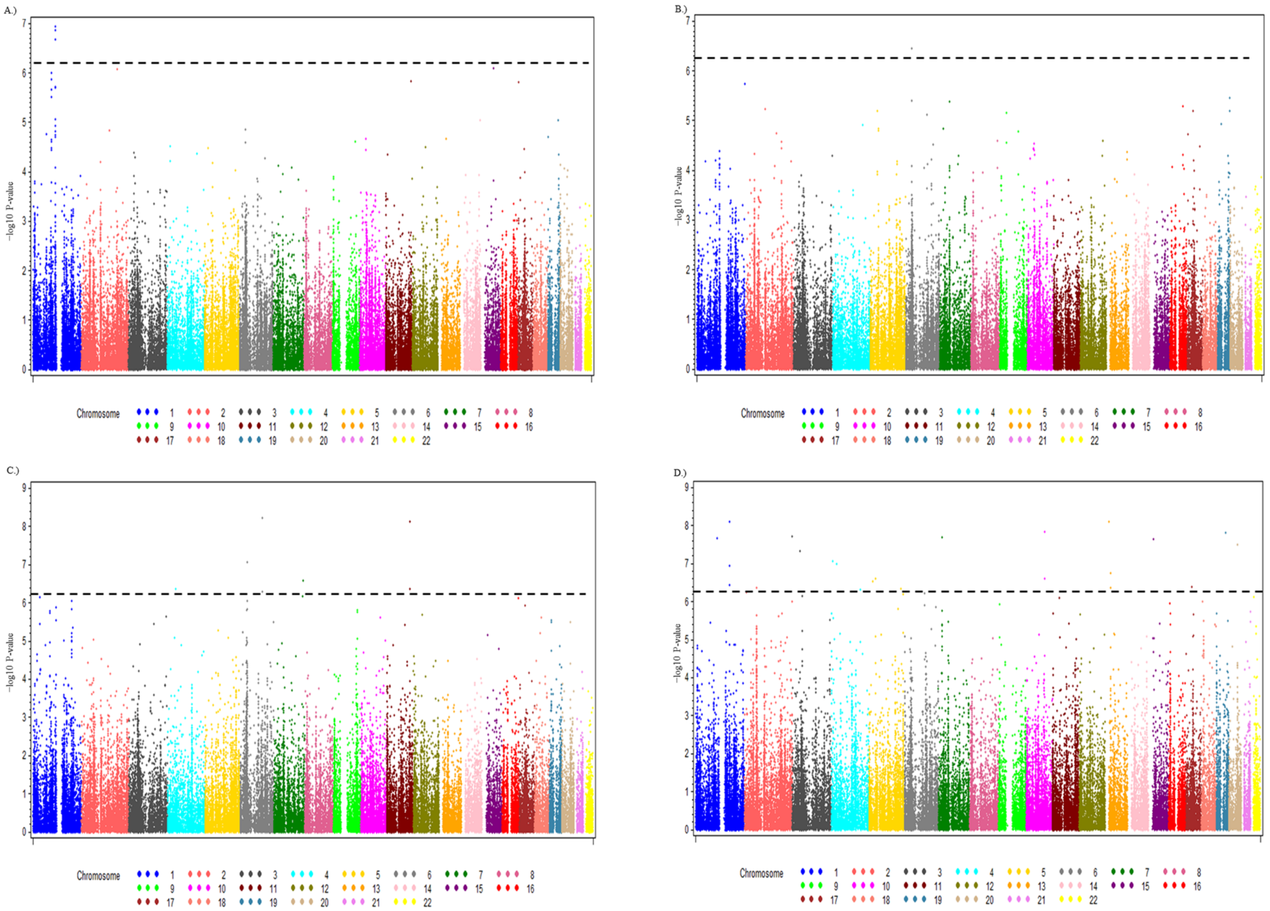

Figure 1. Manhattan plot showing the association P-values of single nucleotide polymorphisms (SNPs) in the ImmunoChip with the time to clearance of HR-HPV. The X-axes display the chromosome on which the SNP is located, the Y-axes display $-\log_{10}$ P-value. The dashed black line represents a significance level needed for multiple testing using the K effective method. Panel A.) Race-adjusted analysis B.) African Americans only C.) European Americans only, and D.) Hispanics only.
doi:10.1371/journal.pone.0099109.g001

A SNP (rs1633038) located on chromosome 6 in the *HLA-G* gene region, was significantly associated with higher clearance rates (HR = 1.89, p = 3.48×10^{-07}) in African Americans, although this was not observed in Hispanics or European Americans separately. Several other SNPs presented in Table 1 were significantly associated with clearance; however, to date, biological relevance of these SNPs and HPV is unclear.

Discussion

We report several variants in immune related genes that are associated with clearance of HR-HPV infection in African Americans, Hispanics, and European Americans HIV-positive women after accounting for the effects of CD4+ count, other LR-HPV co-infection(s), population stratification, and genotyping facility. In particular, CD4+ has been a major factor with HPV persistence/clearance among HIV patients; thus it was adjusted as a time-varying covariate. Also, none of the top hits in table 1 was associated with CD4+ change over time of infection, suggesting that these are independent SNP associations. The most significant association with time to clearance of HR-HPV in the adjusted analysis was seen with several SNPs located on chromosome 1 within the *MAGI3* gene region: rs1112085, rs11102637, and rs12030900 (in LD). The minor alleles for these SNPs in LD were associated with faster time (days) to clearance. MAGI-3 is part of the membrane-associated guanylate kinases (MAGUK) family of proteins that have inverted domain structure, and are part of the PDZ domain-containing proteins, which are localized between epithelial cells [30,31]. The E6 protein of HPV inhibits cellular apoptosis or growth arrest [32]. Several *in vivo* studies have shown that HR-HPV E6 proteins target MAGI-1,-2,-3 proteins for degradation [30,31,33]. This degradation process appears to be necessary for cell transformation [31,34]. Women in our cohort that had the minor allele for the SNPs in the intragenic region of MAGI-3 cleared the HPV infection faster than those with the major allele, and we hypothesize that these genetic variants may interact with HPV differently and therefore affect time to clearance.

Another interesting finding was that the minor allele for SNP rs8031627 located on chromosome 15 in the *SMAD3* gene was significantly associated with higher clearance rates in the adjusted analysis. SMAD proteins are signal transducers and transcriptional modulators that mediate multiple signaling pathways including TGF-β signals [35,36], which inhibits the proliferation of most epithelial cells [36]. The E7 protein of HPV has been shown *in vivo* to block SMAD3 by binding to its target sequence of DNA, which then also inhibits TFG-β for inhibiting DNA synthesis [36]. Again,

Table 1. Cox proportional Hazard Ratios (HR) for the SNPS associated with time to clearance of high-risk (HR-HPV) HPV infection in the race-adjusted analysis, individual race-specific analysis, and pooled analysis of the three races separately.

				Race-Adjusted		Hispanics		African Americans		European Americans		Pooled Analysis*	
**SNP**	**chr**	**position**	**Gene**	**HR**	**p**	**HR**	**P**	**HR**	**p**	**HR**	**p**	**HR**	**p**
rs1112085	1	113742688	*MAGI3*	1.51	1.14E-07	0.98	0.91	1.49	4.07E-05	1.53	0.01	1.33	0.03
rs11102637	1	113793315	*MAGI3*	1.51	1.34E-07	0.98	0.91	1.47	5.78E-05	1.53	0.01	1.33	0.03
rs12030900	1	113775786	*MAGI3*	1.50	2.11E-07	0.98	0.91	1.47	7.49E-05	1.53	0.01	1.32	0.03
rs8031627	15	65271173	*SMAD3*	1.53	8.04E-07	1.76	0.0005	1.44	0.003	1.34	0.08	1.49	1.78E-06
rs1633038	6	29848016	*HLA-G*	1.61	2.5E-05	0.79	0.43	1.89	3.48E-07	1.35	0.20	1.34	0.22
rs1125341	6	34894283	*UHRF1BP1*	0.85	0.01	0.96	0.86	0.93	0.36	1.77	8.41E-08	1.18	0.51
rs180327	11	116128869	*BUD13*	1.16	0.02	0.89	0.48	1.05	0.62	0.50	7.52E-09	0.77	0.30
rs619054	11	116166023	*APOA5*	0.85	0.03	1.03	0.92	1.03	0.74	0.52	4.26E-07	0.81	0.41
rs10952259	7	149764996	*GIMAP8*	1.14	0.03	1.62	0.04	1.01	0.86	1.81	2.68E-07	1.42	0.12
rs16852584	4	40582748	*APBB2*	1.10	0.45	0.53	0.05	1.06	0.69	3.36	4.3E-07	1.25	0.63
rs17511504**	6	111737220	*LOC100128477*							4.33	6.07E-09		
rs117611750**	6	111892918	*REV3L*							4.13	5.11E-07		
rs74833651**	6	111894441	*REV3L*							4.13	5.11E-07		

*using meta-analysis approach;
**informative only in European Americans (e.g. MAF).
doi:10.1371/journal.pone.0099109.t001

women in our cohort that had the minor allele for the SNP in UTR of SMAD3 cleared the HPV infection faster than those with the major allele. We hypothesize that the major allele variant may interact with HPV differently and therefore increase time to clearance.

Interestingly, the most significant association with time to clearance of HR-HPV in the African Americans analysis was with the SNP rs1633038 located on chromosome 6. The minor allele for SNP rs1633038 was associated with faster time (days) to clearance 1.9 times ($HR = 1.89$, $p = 3.48 \times 10^{-7}$) in that those that were homozygous for the minor allele had faster clearance compared to heterozygous and homozygous for the major allele. This SNP is located in chromosome 6 near the HLA-G gene region. HLA-G is a nonclassical HLA class Ib molecule that regulates the immune response through interaction with surface receptors on natural killer, T and antigen-presenting cells [37–40]. Several studies have reported associations between HLA-G polymorphisms and HPV infection susceptibility and persistence [39–41]. A 14 base pair deletion in the 3′ of HLA has also been shown to promote high-risk HPV infection and invasive cervical cancer in various populations [42–44]. In our study we report an association between a SNP near the HLA-G region and HPV clearance; however, specific HLA-G alleles could not be determined through the available data in this region. While there were some differences in minor allele frequency of the variants in table 1 among the three races, there did not seem to be any correlation with the strength of direction of the associations. Further research is warranted to validate these findings and to determine the function of the SNP or if it is in LD with known SNPs in other studies.

We limited our analysis to clearance of HR-HPV infections since these types are most likely to be associated with cervical cancer risk. The underlying hypothesis with this approach is that the biology and pathogenesis of cancer-causing HR-HPV infections should be similar in relation to the host. While the model adjusted for the correlation between the HPV types, it did not produce hazard ratios specific to each HPV type. We were underpowered to assess HPV types separately; however the HERS cohort is one of the largest HIV-positive cohorts with over four years of HPV follow-up data in the United States. Our sample was comprised of 400 women (258 African Americans, 87 European Americans, and 55 Hispanics), which reduced the statistical power to detect significant findings after adjusting for multiple genetic testing, especially when analyzing the three races separately. However, we were able to observe several significant SNPs in the separate analyses for African Americans and European Americans. The ImmunoChip was designed for use in European populations and could be less informative for other ethnic groups if the disease-associated variants are not shared between them [23]. The sample sizes for European Americans and Hispanics were small, so any race specific associations need further evaluations in larger cohorts. Several SNPs that were significantly associated in multiple races independently seem interesting, even for smaller sample size. Few other SNPs have been shown to be significantly associated with HPV clearance in another cohort of HIV+ adolescents (REACH); however, most were not included in the ImmunoChip and of the few included (e.g. rs228942 and rs9292618) [27], they were not significantly associated with HPV clearance in HERS. REACH comprised of adolescents also at early period of infection and HERS only had adults, mostly after several years of infection and thus may indicate different network of immune-related genes involved in the two scenarios and will need caution in interpretation requiring further research.

Of note, the Q-Q plot (Figure S1 A–D) had high deviation (lamda values of 1.23, 1.49, and 1.57 in European Americans, African Americans and Hispanics, respectively) from the expected line. Caution is needed to interpret these associations as they might also be due to population stratification, repeated measures and the inter-relationships, the Cox proportional hazard model, or the nature of ImmunoChip SNPs that are in high LD within the fine-mapped regions, like MHC on chromosome 6 [45,46]. Women with multiple HPV infections were included in the Cox proportional hazard model with the WLW extension, which should account for the correlation between the individuals' data being used multiple times, but this could have an effect on the Q-Q plot due to the population substructure. The ImmunoChip has dense coverage of the MHC region as well as other regions so this may explain the deviation. Since the SNPs are close together, they are in high LD and therefore result in similar p-values. To our knowledge, the majority of genetic studies model the association using logistic regression, and the same assumptions of the Q-Q plot may not hold for the Cox proportional hazard model. The results could reflect a true association since we are assessing the association between a virus and immune related genes; therefore, we would expect a complex network of genes to play a role in clearance of HR-HPV and our significant findings are biologically plausible. Such an approach and methods have not been used often, specifically with high density SNP analyses; thus, it is difficult to interpret the actual reason of the observed deviations.

This analysis assessing SNPs in immune related genes and their associations with HR-HPV clearance brought forth hypotheses regarding several significant SNPs and gene regions. While these SNPs are associated with HPV clearance, future studies could examine if they are also associated with progression of pre-cancer neoplasia in larger cohorts with adequate events during follow-up periods. The variants in the current ImmunoChip are based on a consortium of genes involved in autoimmune diseases, and do not represent the comprehensive genes involved in human immunity. However, future research is needed to validate these associations and finemap the gene regions (which are not as dense in ImmunoChip for most regions) to identify one or multiple rare variants in LD with the functionally associated SNPs in ImmunoChip, specifically with *MAGI-3, SMAD3 and HLA-G* or other biologically plausible genes, potentially involved with HPV clearance.

Supporting Information

Figure S1 Quantile-quantile (Q-Q) plot showing the association P-values of single nucleotide polymorphisms (SNPs) in the ImmunoChip with the time to clearance of HR-HPV. The X-axes display the expected $-\log_{10}$ P-value, the Y-axes display the observed $-\log_{10}$ P-value.
(TIF)

Table S1 Results of Cox Proportional Hazard Ratios (HR) associated with time to clearance of high-risk (HR-HPV) HPV infection for all the SNPs in the ImmunoChip in the race-adjusted analysis (Adujsted), individual race-specific analysis (EA = European American, AA = African American, HIS = Hispanic), and pooled analysis (Meta Analysis) of the three races separately.
(TXT)

Acknowledgments

We thank Dr. Michael S Saag, Dr. Molly Bray, Dr. Emily Levitan and Dr. Hemant Tiwari for their insights with study design, analysis and interpretation of the data. The authors especially thank HERS participants and the HERS Research Group, which consists of: Robert S. Klein, M.D.,

Ellie Schoenbaum, M.D., Julia Arnsten, M.D., M.P.H., Robert D. Burk, M.D., Chee Jen Chang, Ph.D., Penelope Demas, Ph.D., and Andrea Howard, M.D., M.Sc., from Montefiore Medical Center and the Albert Einstein College of Medicine; Jack Sobel, M.D. from the Wayne State University School of Medicine; Anne Rompalo, M.D., David Vlahov, Ph.D., Keerti Shah, M.D, and David Celentano, Ph.D., from the Johns Hopkins University School of Medicine; Charles Carpenter, M.D., Susan Cu-Uvin, MD, Joseph Hogan, ScD, and Kenneth Mayer, M.D. from the Brown University School of Medicine; Ann Duerr, M.D., Lytt I. Gardner, Ph.D., Charles M. Heilig, PhD., Scott Holmberg, M.D., Denise Jamieson, M.D., Caroline C. King, Ph.D., Jan Moore, Ph.D., Ruby Phelps, B.S., Dawn Smith, M.D., and Dora Warren, Ph.D. from the CDC; and Katherine Davenny, Ph.D. from the National Institute of Drug Abuse.

Disclaimer

The findings and conclusions in this report are those of the authors and do not necessarily represent the official position of the Centers for Disease Control and Prevention and other affiliated institutes.

Author Contributions

Conceived and designed the experiments: SLS CCK AMR SC RSK KVS JDS DJJ SS. Performed the experiments: SLS SS. Analyzed the data: SLS HWW SS. Contributed reagents/materials/analysis tools: HWW CCK DJJ SS. Wrote the paper: SLS SS. Reviewed and edited the manuscript: HWW CCK AMR SC RSK KVS JDS DJJ.

References

1. Sudenga SL, Royse KE, Shrestha S (2011) Role and uptake of human papillomavirus vaccine in adolescent health in the United States. Adolesc Health Med Ther 2011: 63–74.
2. Bosch FX (2003) Epidemiology of human papillomavirus infections: new options for cervical cancer prevention. Salud Publica Mex 45 Suppl 3: S326–339.
3. Bosch FX, de Sanjose S (2002) Human papillomavirus in cervical cancer. Curr Oncol Rep 4: 175–183.
4. Bosch FX, Lorincz A, Munoz N, Meijer CJ, Shah KV (2002) The causal relation between human papillomavirus and cervical cancer. J Clin Pathol 55: 244–265.
5. Ferlay J, Shin HR, Bray F, Forman D, Mathers C, et al. (2010) Estimates of worldwide burden of cancer in 2008: GLOBOCAN 2008. Int J Cancer 127: 2893–2917.
6. Ho GY, Bierman R, Beardsley L, Chang CJ, Burk RD (1998) Natural history of cervicovaginal papillomavirus infection in young women. N Engl J Med 338: 423–428.
7. Moscicki AB, Shiboski S, Broering J, Powell K, Clayton L, et al. (1998) The natural history of human papillomavirus infection as measured by repeated DNA testing in adolescent and young women. J Pediatr 132: 277–284.
8. Sudenga SL, Shrestha S (2013) Key considerations and current perspectives of epidemiological studies on human papillomavirus persistence, the intermediate phenotype to cervical cancer. Int J Infect Dis.
9. Shi Y, Li L, Hu Z, Li S, Wang S, et al. (2013) A genome-wide association study identifies two new cervical cancer susceptibility loci at 4q12 and 17q12. Nat Genet 45: 918–922.
10. Chen D, Juko-Pecirep I, Hammer J, Ivansson E, Enroth S, et al. (2013) Genome-wide association study of susceptibility loci for cervical cancer. J Natl Cancer Inst 105: 624–633.
11. Demeret C, Le Moal M, Yaniv M, Thierry F (1995) Control of HPV 18 DNA replication by cellular and viral transcription factors. Nucleic Acids Res 23: 4777–4784.
12. Dong XP, Stubenrauch F, Beyer-Finkler E, Pfister H (1994) Prevalence of deletions of YY1-binding sites in episomal HPV 16 DNA from cervical cancers. Int J Cancer 58: 803–808.
13. Tindle RW (2002) Immune evasion in human papillomavirus-associated cervical cancer. Nat Rev Cancer 2: 59–65.
14. O'Brien PM, Campo MS (2003) Papillomaviruses: a correlation between immune evasion and oncogenicity? Trends Microbiol 11: 300–305.
15. Hildesheim A, Schiffman M, Scott DR, Marti D, Kissner T, et al. (1998) Human leukocyte antigen class I/II alleles and development of human papillomavirus-related cervical neoplasia: results from a case-control study conducted in the United States. Cancer Epidemiol Biomarkers Prev 7: 1035–1041.
16. Lee SJ, Cho YS, Cho MC, Shim JH, Lee KA, et al. (2001) Both E6 and E7 oncoproteins of human papillomavirus 16 inhibit IL-18-induced IFN-gamma production in human peripheral blood mononuclear and NK cells. J Immunol 167: 497–504.
17. Stanley M (2006) Immune responses to human papillomavirus. Vaccine 24 Suppl 1: S16–22.
18. Hildesheim A, Wang SS (2002) Host and viral genetics and risk of cervical cancer: a review. Virus Res 89: 229–240.
19. Mahmud SM, Robinson K, Richardson H, Tellier PP, Ferenczy AS, et al. (2007) HLA polymorphisms and cervical human Papillomavirus infection in a cohort of Montreal University students. J Infect Dis 196: 82–90.
20. Koshiol JE, Schroeder JC, Jamieson DJ, Marshall SW, Duerr A, et al. (2006) Time to clearance of human papillomavirus infection by type and human immunodeficiency virus serostatus. Int J Cancer 119: 1623–1629.
21. Smith DK, Warren DL, Vlahov D, Schuman P, Stein MD, et al. (1997) Design and baseline participant characteristics of the Human Immunodeficiency Virus Epidemiology Research (HER) Study: a prospective cohort study of human immunodeficiency virus infection in US women. Am J Epidemiol 146: 459–469.
22. Cortes A, Brown MA (2011) Promise and pitfalls of the Immunochip. Arthritis Res Ther 13: 101.
23. Parkes M, Cortes A, van Heel DA, Brown MA (2013) Genetic insights into common pathways and complex relationships among immune-mediated diseases. Nat Rev Genet 14: 661–673.
24. Manichaikul A, Mychaleckyj JC, Rich SS, Daly K, Sale M, et al. (2010) Robust relationship inference in genome-wide association studies. Bioinformatics 26: 2867–2873.
25. Moskvina V, Schmidt KM (2008) On multiple-testing correction in genome-wide association studies. Genet Epidemiol 32: 567–573.
26. Xue X, Gange SJ, Zhong Y, Burk RD, Minkoff H, et al. (2010) Marginal and mixed-effects models in the analysis of human papillomavirus natural history data. Cancer Epidemiol Biomarkers Prev 19: 159–169.
27. Sudenga SL, Wiener HW, Shendre A, Wilson CM, Tang J, et al. (2013) Variants in interleukin family of cytokines genes influence clearance of high risk HPV in HIV-1 coinfected African-American adolescents. Hum Immunol 74: 1696–1700.
28. Price AL, Patterson NJ, Plenge RM, Weinblatt ME, Shadick NA, et al. (2006) Principal components analysis corrects for stratification in genome-wide association studies. Nat Genet 38: 904–909.
29. Purcell S, Neale B, Todd-Brown K, Thomas L, Ferreira MA, et al. (2007) PLINK: a tool set for whole-genome association and population-based linkage analyses. Am J Hum Gen 81: 559–575.
30. Thomas M, Laura R, Hepner K, Guccione E, Sawyers C, et al. (2002) Oncogenic human papillomavirus E6 proteins target the MAGI-2 and MAGI-3 proteins for degradation. Oncogene 21: 5088–5096.
31. Ainsworth J, Thomas M, Banks L, Coutlee F, Matlashewski G (2008) Comparison of p53 and the PDZ domain containing protein MAGI-3 regulation by the E6 protein from high-risk human papillomaviruses. Virol Journal 5: 67.
32. Doorbar J, Quint W, Banks L, Bravo IG, Stoler M, et al. (2012) The biology and life-cycle of human papillomaviruses. Vaccine 30 Suppl 5: F55–70.
33. Glaunsinger BA, Lee SS, Thomas M, Banks L, Javier R (2000) Interactions of the PDZ-protein MAGI-1 with adenovirus E4-ORF1 and high-risk papillomavirus E6 oncoproteins. Oncogene 19: 5270–5280.
34. Simonson SJ, Difilippantonio MJ, Lambert PF (2005) Two distinct activities contribute to human papillomavirus 16 E6's oncogenic potential. Cancer Res 65: 8266–8273.
35. Massague J, Wotton D (2000) Transcriptional control by the TGF-beta/Smad signaling system. The EMBO Journal 19: 1745–1754.
36. Lee DK, Kim BC, Kim IY, Cho EA, Satterwhite DJ, et al. (2002) The human papilloma virus E7 oncoprotein inhibits transforming growth factor-beta signaling by blocking binding of the Smad complex to its target sequence. J Biol Chem 277: 38557–38564.
37. Carosella ED, Favier B, Rouas-Freiss N, Moreau P, Lemaoult J (2008) Beyond the increasing complexity of the immunomodulatory HLA-G molecule. Blood 111: 4862–4870.
38. Simoes RT, Goncalves MA, Castelli EC, Junior CM, Bettini JS, et al. (2009) HLA-G polymorphisms in women with squamous intraepithelial lesions harboring human papillomavirus. Mod Pathol 22: 1075–1082.
39. Ferguson R, Ramanakumar AV, Koushik A, Coutlee F, Franco E, et al. (2012) Human leukocyte antigen G polymorphism is associated with an increased risk of invasive cancer of the uterine cervix. Int J Cancer 131: E312–319.
40. Ferguson R, Ramanakumar AV, Richardson H, Tellier PP, Coutlee F, et al. (2011) Human leukocyte antigen (HLA)-E and HLA-G polymorphisms in human papillomavirus infection susceptibility and persistence. Hum Immunol 72: 337–341.
41. Metcalfe S, Roger M, Faucher MC, Coutlee F, Franco EL, et al. (2013) The association between human leukocyte antigen (HLA)-G polymorphisms and human papillomavirus (HPV) infection in Inuit women of northern Quebec. Hum Immunol 74: 1610–1615.
42. Bortolotti D, Gentili V, Rotola A, Di Luca D, Rizzo R (2014) Implication of HLA-G 3′ untranslated region polymorphisms in human papillomavirus infection. Tissue Antigens 83: 113–118.
43. Yang YC, Chang TY, Chen TC, Lin WS, Chang SC, et al. (2014) Human leucocyte antigen-G polymorphisms are associated with cervical squamous cell carcinoma risk in Taiwanese women. Eur J Cancer 50: 469–474.
44. Silva ID, Muniz YC, Sousa MC, Silva KR, Castelli EC, et al. (2013) HLA-G 3′UTR polymorphisms in high grade and invasive cervico-vaginal cancer. Hum Immunol 74: 452–458.
45. Pearson TA, Manolio TA (2008) How to interpret a genome-wide association study. JAMA 299: 1335–1344.

46. Juran BD, Hirschfield GM, Invernizzi P, Atkinson EJ, Li Y, et al. (2012) Immunochip analyses identify a novel risk locus for primary biliary cirrhosis at 13q14, multiple independent associations at four established risk loci and epistasis between 1p31 and 7q32 risk variants. Hum Mol Genet 21: 5209–5221.
